# Positive influence of long-term growth hormone therapy on circulating stem cells in pediatric patients with growth hormone deficiency

**DOI:** 10.1038/s41598-025-20532-6

**Published:** 2025-10-17

**Authors:** Anna Wędrychowicz, Katarzyna Sielatycka,, Magda Kucia, Ewa Kubiś, Dorota Roztoczyńska, Jerzy B. Starzyk

**Affiliations:** 1 Department of Pediatric and Adolescent Endocrinology, Pediatric Institute, Jagiellonian University Medical College, Krakow, Poland; 2 Department of Pediatric and Adolescent Endocrinology, University Children’s Hospital in Krakow, Wielicka 265, 30-663 Cracow, Poland; 3 Institute of Biology, Faculty of Exact and Natural Sciences, University of Szczecin, ul. Felczaka 3c, 71-415 Szczecin, Poland; 4 Sanprobi Sp. z.o.o. Sp.k., ul. Kurza Stopka 5C, 70-535 Szczecin, Poland; 5 Department of Regenerative Medicine, Medical University of Warsaw, Warsaw, Poland; 6 Department of Physiology, Pomeranian Medical University in Szczecin, Szczecin, Poland

**Keywords:** Very small embryonic/epiblast‐like stem cells (VSELs), Mesenchymal stromal cells (MSCs), Endothelial progenitor cells (EPCs), Insulin-like growth factor 1 (IGF-1), Growth hormone (GH), Pediatric patients with growth hormone deficiency, Developmental biology, Stem cells, Endocrinology

## Abstract

Very small embryonic/epiblast‐like stem cells (VSELs), present in circulating blood, are small, non-hematopoietic cells that express markers of pluripotent embryonic and primordial germ cells. VSELs are believed to contribute to postnatal tissue and organ regeneration. We report the first long-term observation of VSELs in response to growth hormone (GH) therapy in pediatric patients. Twenty patients aged 5.2–13.4 years with GH-deficiency were monitored periodically during the first year of GH treatment. Eight of these patients were re-examined after eight years of continuous therapy. Selected stem cell populations were analyzed in peripheral blood using flow cytometry. Long-term GH therapy resulted in increased numbers of CD34 + VSELs, while CD133 + VSELs remained comparable to baseline. The increase in VSELs was accompanied by parallel increases in circulating hematopoietic stem cells, mesenchymal stromal cells (MSCs), and endothelial progenitor cells. We observed significant positive correlations between VSELs and MSCs, and between CD34 + VSELs and MSCs with postprandial glucose levels. These data suggest that VSELs are responsive to GH treatment. Long-term GH therapy appears to modulate VSEL populations without causing harm and may even enhance them in GH-deficient patients. Unlike findings in experimental animals, GH therapy in humans did not show adverse effects on lifespan or organ function.

## Introduction

Currently, the effects of growth hormone (GH) on aging and life expectancy have still been controversial. GH offers several benefits beyond its immediate impact on growth, including increased exercise performance, bone density, and muscle mass, as well as reduced body fat^1,2^. These effects make GH of interest in anti-aging medicine^3,4^. Indeed, a significantly reduced lifespan has been observed in patients with hereditary, untreated growth hormone deficiency (GHD) due to GH-1 gene depletion^5^. However, other data suggest that untreated GHD does not affect longevity in humans^6,7^.

Clinical observations show that high levels of GH may accelerate aging and increase the risk of cardiovascular disorders. Individuals with untreated acromegaly, resulting from excess GH, have a significantly shorter life expectancy compared to the healthy population^8^, primarily due to metabolic complications and increased cancer risk. Conversely, long-term GHD is associated with decreased well-being, anxiety and/or depression, reduced energy levels, increased body fat, and insulin resistance, which can lead to type 2 diabetes. It is also linked to decreased muscle tone, reduced bone density leading to osteoporosis, and elevated LDL-cholesterol and triglyceride levels, all of which contribute to higher cardiovascular risk^9^. Patients with Prader-Willi syndrome and those with chronic renal disease not only experience significant improvements in final adult height but also show multiple metabolic benefits during recombinant human GH (rhGH) therapy^10^. Moreover, clinical observations indicate that long-term rhGH therapy is not associated with an increased risk of carcinogenesis or cardiometabolic disorders, even in patients who have undergone oncological treatments^11–13^.

The mechanism responsible for the participation of GH in aging could include the GH/Insulin/Insulin-like growth factor-1 (GH/INS/IGF-1) signaling action on very small embryonic/epiblast‐like stem cells (VSELs). VSELs, which exist in mouse and human bone marrow and other tissues, are cells presenting with markers of pluripotent embryonic and primordial germ cells^14,15^. These unique cells, which are somewhat smaller than red blood cells^16^, (1) mobilize in the peripheral blood during stressful situations^17^, (2) are in mice embellished with the fraction of Sca1 + Lin-CD45- cells and in humans with the fraction of (CD133 + /CD34 +)Lin-CD45- cells, (3) express pluripotent stem cell markers (e.g., Oct-4, Nanog, and SSEA), characterizing cells less differentiated than bone marrow-derived mesenchymal stromal cells (MSCs)^18^, and (4) exhibit a definite morphology with a high ratio of nucleus-to-cytoplasm and unvarying chromatin. VSELs are considered precursors of long-term repopulating hematopoietic stem cells (HSCs), mesenchymal stromal cells (MSCs), and endothelial progenitor cells (EPCs)^19^. There is a positive correlation between the number of these cells in adult tissues and life expectancy^20^. VSELs are nominated as a backup population for adult tissue-committed stem cells (TCSCs). Therefore VSELs are considered to be responsible for the rejuvenation of tissues and organs and it is reasonable to assume that their strength and general state affect life expectancy^20^. The potential of endogenous, pluripotent VSELs offers a promising approach in a three-front strategy against tissue damage, cancer, and aging^21^. However, the first observations in animal models have shown that their number could decrease after long-term administration of GH or IGF-1^22^. These observations have raised concerns about the future of patients treated with rhGH. Moreover it is known that long-term replacement of GH in high doses, reduces insulin sensitivity and exacerbates insulin resistance. VSEL counts have been shown to be correlated with lifespan. Furthermore, restriction of calories, regular exercise and metformin improving insulin sensitivity positively affect maintaining VSELs in adult tissues^22^.

Therefore, this study aimed to investigate the effects of recombinant human GH (rhGH) therapy on circulating stem cell populations in peripheral blood and their correlations with metabolic parameters. Based on long-term clinical observations, we hypothesize that rhGH therapy does not negatively affect the stem cell pool or metabolic outcomes in pediatric patients with GH deficiency receiving this essential replacement therapy.

## Methods

### Patients

We conducted a cohort study in 20 consecutive patients with isolated idiopathic GHD who met the national eligibility criteria for rhGH therapy in 2014. Patients were aged 5.04–13.37 years (mean ± SD, 9.1 ± 2.7 years). Before the rhGH therapy, the mean standard deviation (SD) of patients’ height was − 3.35 ± 0.75. The mean rhGH dose was 0.27 mg/kg/week.

The study was approved by the Local Ethical Committee (No. KBET/14/B/2014 from 27 February 2014 and No. 1072.6120.114.2022 from 31 August 2022). All research was performed in accordance with relevant regulations and the Declaration of Helsinki. Written informed consent was obtained from all participants and/or their legal guardians.

Fasting blood samples were collected prior to rhGH treatment to measure complete blood counts and target parameters. Follow-up samples were collected at 2 weeks, 1, 3, and 6 months thereafter. We assessed fluctuations in the numbers of circulating small cells, including very small embryonic-like stem cells (VSELs; CD133 + Lin − CD45 − and CD34 + Lin − CD45 −), hematopoietic stem cells (HSCs; CD133 + Lin − CD45 + and CD34 + Lin − CD45 +), mesenchymal stromal cells (MSCs), and endothelial progenitor cells (EPCs).

Serum insulin-like growth factor 1 (IGF-1) levels were measured before rhGH therapy and at 3 and 6 months. One year after therapy initiation, serum IGF-1, glucose, and insulin levels during an oral glucose tolerance test (OGTT) were measured. After eight years, we repeated the analysis in eight patients who continued rhGH therapy. At that time, the mean age of these patients was 14.1 ± 1.1 years. The remaining 12 patients had completed therapy according to our national program criteria. In the eight patients still treated, fasting blood samples were analyzed for blood count, VSELs, HSCs, and EPCs. Serum IGF-1 was measured and OGTT were also performed.

### Flow cytometry

Peripheral blood (PB) samples were subjected to double lysis with BD Pharm Lyse buffer (BD Biosciences) for 10 min at room temperature. After lysis, samples were washed with phosphate-buffered saline (PBS) containing 2% fetal bovine serum (FBS; Sigma Aldrich) to obtain the total nucleated cell (TNC) count, as described previously^23^. TNCs were stained in PBS with 2% FBS for 30 min on ice, washed, and resuspended. Flow cytometric analysis was performed on a Navios flow cytometer (Beckman Coulter), with at least 1 × 10⁶ events acquired per sample. For each patient, the absolute numbers of very small embryonic-like stem cells (VSELs) and white blood cells (WBCs) per 1 μL of PB were calculated based on the percentages obtained by flow cytometry. Data were analyzed using Kaluza software (Beckman Coulter).

### Analysis of circulating stem and progenitor cells

To identify very small embryonic-like stem cells (VSELs) and hematopoietic stem cells (HSCs), TNCs were stained with a cocktail of fluorescein isothiocyanate (FITC)-conjugated antibodies against hematopoietic lineage markers (Lin: CD2, CD3, CD14, CD16, CD19, CD24, CD56, CD66b, CD235a; BD Biosciences). In parallel, cells were labeled with phycoerythrin (PE)-conjugated anti-CD45 antibody (clone HI30, BD Biosciences) and either allophycocyanin (APC)-conjugated anti-CD34 (clone 581, BD Biosciences) or APC-conjugated anti-CD133/1 (Miltenyi Biotec). VSELs were defined as Lin − /CD45 − /CD34 + or CD133 + cells. These cells are very small, typically less than 6 μm in diameter, and their size was carefully considered during flow cytometric analysis using forward scatter (FSC) parameters to distinguish them from larger hematopoietic cells, ensuring accurate identification of the VSEL population. HSCs were defined as Lin − /CD45 + /CD34 + or CD133 + cells. HSC are generally larger than VSELs, typically ranging from 8–12 μm in diameter, which was taken into account during flow cytometric analysis.

Endothelial progenitor cells (EPCs) were identified as CD34 + /CD133 + /KDR + using antibodies against CD45 (FITC, clone HI3; BD Biosciences), CD133 (APC, clone CD133/1; Miltenyi Biotec), CD34 (PE-Cy7, clone 4H11; BD Biosciences), and KDR/VEGFR2 (PE, clone 89106; R&D Systems).

Mesenchymal stromal cells (MSCs) were defined as CD45 − /CD105 + /CD90 + /CD29 + using antibodies against CD45 (FITC, clone HI3; BD Biosciences), CD105 (APC, clone 43A3; BioLegend), CD90 (PE, clone 5E10; BioLegend), and CD29 (PE, clone MAR4; BD Biosciences).

Matched isotype controls were included in all experiments: FITC-, PE-, and APC-conjugated mouse IgG1 (clone MOPC-21, BD Biosciences), IgG2a (clone G155-178, BD Biosciences), IgG2b (clone 27–35, BD Biosciences), and APC-conjugated IgG1 (clone IS5-21F5, Miltenyi Biotec).

Antibody incubations were performed for 30 min on ice, followed by washing and resuspension. Samples were analyzed on a Navios flow cytometer (Beckman Coulter) with ≥ 1 × 10⁶ events per sample. Data were processed using Kaluza software (Beckman Coulter).

### Biochemical tests

Blood counts were measured using the Vitros 950 (Ortho Clinical Diagnostics, Rochester, NY, USA). IGF-1 and insulin were measured by immunochemistry: IGF-1 (Diasource, detection limit 8.8 ng/mL; intra-assay precision < 9.8%, inter-assay precision < 10.4%) and insulin (Siemens Diagnostics, detection limit 0.5 mU/L; intra-assay precision ≤ 4.6%, inter-assay precision ≤ 5.9%).

### Statistical analysis

The Staf Soft Statistica 12 package was used to perform the statistical analysis. An ANOVA with a post-hoc Tukey’s test and linear and multivariate regression were performed for the analysis. The Spearman correlation coefficient was applied. We assumed that a *p* < 0.05 is statistically significant.

## Results

Administration of rhGH had a significant impact on the number of very small embryonic-like stem cells (VSELs) in peripheral blood. During the first three months of therapy, VSEL numbers increased, followed by a decline at six months, reaching levels below baseline. Interestingly, after eight years of continuous rhGH treatment, VSEL numbers increased again. Specifically, long-term therapy resulted in elevated CD34 + VSEL counts compared with baseline, while CD133 + VSELs remained comparable to baseline values.

**Fig. 1:**
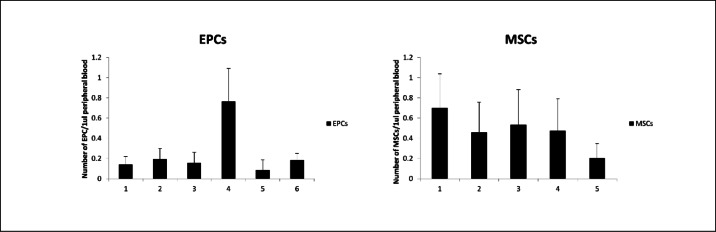
Mean ± SD values of circulating CD34⁺ very small embryonic-like stem cells (VSELs)/hematopoietic stem cells (HSCs) (left panel) and CD133⁺ VSELs/HSCs (right panel) in peripheral blood at six time points: (1) baseline (before initiation of recombinant human growth hormone, rhGH), (2) 2 weeks, (3) 1 month, (4) 3 months, (5) 6 months, and (6) 8 years after rhGH introduction. Data are expressed as absolute numbers of cells per μL of peripheral blood.

**Fig. 2:**
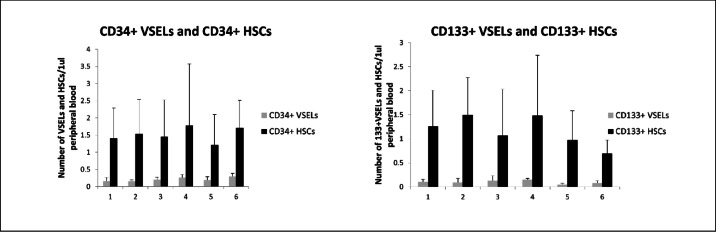
Mean ± SD values of circulating endothelial progenitor cells (EPCs) (left panel) and mesenchymal stromal cells (MSCs) (right panel) in peripheral blood at six time points: (1) baseline (before initiation of recombinant human growth hormone, rhGH), (2) 2 weeks, (3) 1 month, (4) 3 months, (5) 6 months, and (6) 8 years after rhGH introduction. Data are expressed as absolute numbers of cells per μL of peripheral blood.

The increase in VSELs was accompanied by parallel changes in circulating mesenchymal stromal cells (MSCs) and endothelial progenitor cells (EPCs). After eight years, EPC numbers were comparable to baseline. MSCs could not be assessed in the long-term, though they decreased after six months relative to pre-treatment values. Temporal changes in CD34 + and CD133 + VSELs, HSCs, MSCs, and EPCs are presented in Figs. 1 and 2.

In statistical analysis, significant positive correlations were found between CD34 + VSELs and CD133 + VSELs (r = 0.285, *p* = 0.01), between CD34 + VSEls and CD34 + HSCs (r = 0.238, *p* = 0.03) and between CD133 + VSELs and CD133 + HSCs (r = 278, *p* = 0.01). There was also strong positive correlation between CD34 + HSCs and CD133 + HSCs (r = 0.87, *p* = 0.03). We found a significant, positive correlation between VSELs and MSCs (for CD34 + VSELs vs MSCs r = 0.33, *p* < 0.01; and for CD133 + VSELs vs MSCs r = 0.697, *p* < 0.01), but none correlation between HSCs and MSCs.

Simultaneously we observed significant changes of IGF-1 levels during rhGH treatment (*p* = 0.001) (Table 1). Before therapy IGF-1 levels were low, with a mean SD value of − 1.5 ± 1.1, then there was observed a gradual increase of it up to + 0.3 ± 2.0 after six months and up to + 1.6 ± 2.1 after one year of the therapy. In long-term observation the IGF-1 level was still within normal ranges with mean SD + 1.2 ± 1.0 after eight years of rhGH therapy. We did not find any correlation between IGF-1 and VSELs, MSCs nor EPCs levels three, six, one year and eight years after rhGH introduction.

**Table 1:** The mean data ± SD of IGF-1 levels and the height’s parameters in the participants of the study during therapy with recombinant human growth hormone (rhGH) SDS – standard deviation sore.

	Before rhGH therapy n = 20	After 3 months of rhGH therapy n = 20	After 1 yr of rhGH therapy n = 20	After 8 yrs of rhGH therapy n = 8	*P* value ANOVA post hoc Tukey
SDS of IGF-1	− 1.7 ± 0.8	0.2 ± 2.0	1.5 ± 2.2	1.2 ± 1.0	0.001
Height’ SDS	− 3.26 ± 0.75	− 3.11 ± 0.82	− 2.09 ± 0.64	− 0.53 ± 0.38	0.0001

Long-term rhGH therapy also resulted in significant improvements in height (*p* = 0.0001) (Table 1). Notably, height SDs were positively correlated with CD34 + VSEL numbers (r = 0.53, *p* = 0.01) and negatively correlated with CD133 + HSCs (r =  − 0.49, *p* = 0.02). These findings suggest an important role for VSELs in GH-regulated growth, with HSCs contributing to increases in body size.

Glucose and insulin levels, both fasting and postprandial, remained within normal ranges in OGTT, as did HbA1c values, at both one and eight years of therapy (Table 2). Interestingly, MSC numbers were positively correlated with postprandial glucose levels (r = 0.53, *p* = 0.02).

**Table 2:** Metabolic parameters (HbA1c and glucose and insulin in oral glucose tolerance test) during therapy with recombinant human growth hormone (rhGH).

	After 1 yr of rhGH therapy n = 20	After 8 yrs of rhGH therapy n = 8	*P*
Glucose 0 min (mmol/l)	4.7 ± 0.5	4.8 ± 0.3	0.35
Glucose 120 min (mmol/l)	5.4 ± 1.1	5.1 ± 1.0	0.34
Insulin 0 min (mU/l)	10.3 ± 5.8	10.0 ± 2.3	0.80
Insulin 120 min (mU/l)	47.5 ± 40.1	61.8 ± 41.2	0.72
HbA1c (%)	5.3 ± 0.1	5.1 ± 0.1	0.07

## Discussion

For the first time we demonstrate that prolonged treatment with rhGH modulates the population of VSELs, HSCs, MSCs and EPCs circulating in PB of patients with GHD.

VSELs respond dynamically to GH treatment: their numbers in PB significantly increase during the first month of rhGH therapy, followed by a gradual decline over the subsequent six months. Importantly, long-term rhGH therapy does not reduce circulating VSEL levels; rather, it appears to enhance them. VSELs express pluripotent markers, similar to pluripotent stem cells. They can differentiate into cells of all three germ layers and into germ cells^24–26^. Unlike pluripotent stem cells, however, VSELs do not integrate into a developing embryo, do not form teratomas in SCID mice, and do not divide and expand in breeding experiments, although some recent studies report limited success in this regard^27,28^. VSELs have been identified in numerous adult tissues by more than 60 independent research groups^19^. In contrast, human embryonic stem cells and induced pluripotent stem cells exist only in vitro as tissue-culture artifacts, with no direct physiological equivalents in vivo^29^. Despite advances in the use of these cells, their application remains limited due to risks of carcinogenesis, immunogenicity, and heterogeneity. Lines of induced pluripotent stem cells display variable differentiation potential, genomic instability, and abnormal epigenetic patterns^30^. VSELs possess unique characteristics that maintain their dormancy in vivo, thereby preventing spontaneous cancer formation^31,32^. Physiological processes naturally regulate the tumorigenic potential of these pluripotent VSELs. Moreover, recent meta-analyses provide no evidence linking GH replacement therapy to increased cancer-related mortality among childhood cancer survivors^11,13^.

A particularly interesting observation supporting our data is the significant positive correlation between VSELs and HSCs. VSELs display a surface phenotype of LIN– CD45– CD133 + , whereas HSCs exhibit LIN– CD45 + CD133 + in humans, highlighting the heterogeneity between these populations. While CD34 + HSCs were previously considered the most primitive hematopoietic stem cells, recent reports indicate the existence of CD34– HSCs, which redefines the hierarchy and positions VSELs as the most primitive stem cells^33^. A small proportion of LIN– CD45 + HSCs are CD34–, but most express CD34. LIN– CD45– CD34– CD133 + VSELs represent the most primitive hematopoietic stem cells, with pluripotent potential capable of regenerating multiple tissue types. VSELs provide lifelong replacement of tissue progenitors and can be activated to regenerate damaged organs in vivo, including the lungs^34^, pancreas^35^, bone^36^, and endometrial epithelium^37^. Furthermore, similar to natural gamete precursors, VSELs can differentiate into oocytes and sperm in vitro^38^. Under stress conditions in vivo, VSELs may enter the cell cycle; however, the precise mechanisms regulating their quiescence versus proliferation, as well as self-renewal versus differentiation, remain unclear. Similarly, the in vitro conditions required to reliably induce VSEL proliferation and self-renewal have not yet been established. Exposure to growth factors and cytokines can modulate cellular differentiation in vitro, but their epigenetic status largely remains immutable. During long-term follow-up, patients underwent puberty, which could also significantly influence the VSEL population. Notably, steroid and gonadotropin hormone receptors have been identified on VSELs in both humans and mice, suggesting that these cells may respond to hormonal regulation in vivo^39–41^.

The possibility that the observed increase in circulating VSELs reflects mobilization from the bone marrow rather than an actual increase in cell numbers should be considered. Stem cell release, mobilization, and homing are sequential processes with important physiological roles. Previous studies have identified roles for cytokines such as G-CSF and SCF, and adhesion molecules including VLA-4 and P/E selectins, in stem cell mobilization. More recent evidence from experimental animal models and clinical mobilization protocols highlights the involvement of chemokines such as stromal-derived factor-1 (SDF-1) and IL-8, as well as proteolytic enzymes including elastase, cathepsin G, and various MMPs, in the mobilization process^42^. Plasma elevation of SDF-1 has been shown to induce mobilization of both mature and immature hematopoietic progenitor and stem cells^43^. Interestingly, significantly lower levels of SDF-1 have been reported in children with GHD compared to age-matched healthy controls, with a notable increase during the first year of rhGH therapy^44^. This observation suggests that the increase in circulating VSELs during rhGH treatment may result from their mobilization from the bone marrow, potentially mediated by SDF-1.

Moreover, we have found positive relationships between VSELs and HSCs with MSCs. It is very interesting in light of attempts to convert induced pluripotent stem cells into mesenchymal stem/stromal cells^45,46^. An expansion of MSCs can be performed from several sources. Also MSC-derived microvesicles/exosomes present with similar beneficial/regenerative effects after transplantation^47–49^. The beneficial effects of MSC transplantation, as demonstrated in a number of clinical trials^50,51^, are due to their ability to rejuvenate of diseased tissues by paracrine action to VSELs, which then regenerate diseased tissues. The presence of VSELs is necessary to explain the potential of MSCs in the regeneration process. VSELs are responsible for regenerating diseased organs in conditions, where transplanted mesenchymal stromal cells provide paracrine support^52^. MSCs themselves are not pluripotent. As well HSCs as MSCs do not have regenerative potential. They cannot transdifferentiate thus regenerate diseased tissues of other types and lines. HSCs, better defined as "lineage-restricted" and "tissue-committed" progenitors, derived from umbilical cord blood or bone marrow, have had a huge clinical impact on cell regeneration in blood-related diseases^53,54^. In recent years, the possible role of GH in the modulation of MSCs commitment has gained interest. MSCs express GH receptors. GH induces an inhibition of adipogenic differentiation and favors MSC differentiation towards osteogenesis. This activity of GH indicates that regulation of body composition by GH has already started in the tissue progenitor cells^55^.

The administration of GH leads to a gradual increase in IGF-1 levels, raising the question of whether GH acts directly on the VSEL population or indirectly via IGF-1. In our study, no correlations were observed between the populations of VSELs, MSCs, or EPCs and IGF-1 levels, either during the first year of rhGH therapy or in long-term follow-up after eight years. Interestingly, however, we found correlations between HSCs and postprandial glucose in our long-term observations. These findings suggest that GH treatment may influence aging and tissue rejuvenation directly, potentially through insulin signaling rather than via IGF-1. A major strength of our study is the long-term continuous follow-up of patients receiving rhGH therapy.

There was postulated that VSELs are constantly diminished in the “metabolic fire” generated by GH/INS/IGF signaling during adult life^22^. Therefore, the conditions of diminishing this GH/INS/IGF signaling including a restriction of calories intake, regular physical exercise, or some specific drugs decreasing insulin resistance could support longevity^56^. The clinical observation of the population of inactive GH-IGF-1 axis, the Laron dwarfs, with very low level of circulating IGF-1 present with extended life span and lack of malignancies. In detail, patients with Laron Syndrome have an increased risk of developing hyperlipidemia, cardiovascular disease and diabetes mellitus because of severe obesity. Although they present with these metabolic deficits, their longevity is normal. Moreover patients with Laron Syndrome homozygous for GH-receptor defect have no risk of cancer occurrence^57^. Among their relatives, the prevalence of cancer is the same as in general population. In the Ecuadorian cohort of people with Laron Syndrome any case of type 2 diabetes has been reported, whereas its prevalence amounts 5% in Ecuador. In 2013 the first patient with Laron syndrome who developed diabetes mellitus was described, but he was previously treated with recombinant human IGF-1^58^. In 2017, a 57-yr-old Mexican case of a never-treated patient with Laron syndrome who developed metabolic syndrome, type 2 diabetes and stroke was reported^59^. However, the clinical data of this patient deny the recognition of Laron syndrome, because she presented with as well low IGF-1 values as low GH values in the stimulation (with clonidine) test. A defect of GH receptor causes Laron syndrome, therefore patients with this syndrome have very low IGF-1 levels but high GH levels. Therefore, other than ours results reported by Grubczak et al. Their patients with IGF-1 deficiency initially had higher levels of VSEL and HSC compared to healthy ones, with a gradual decline during long-term treatment (4–5 years) with recombinant human IGF-1^60^.

Patients with GHD present with different metabolic complications and have an increased cardiovascular risk and shortened lifetime. This could suggest that excess of GH accompanied by decreased IGF-1 results in longer life expectancy in comparison to a general, healthy population, like it was presented in mice^61^, but low GH and low IGF-1 results in shorter life-expectancy due to metabolic complications, especially in women^5,62^. It was supposed that high GH with high IGF-1 results also with shorter expectancy but due to carcinogenesis^63^. However, long-term clinical follow-up has not confirmed this disturbing report^10,11^. Mutant animals Laron dwarfs with disorder of the gene encoding the GH receptor and the GH binding protein are smaller in size but their longevity is significantly longer than their normal siblings, present with delayed aging, and have low serum levels of IGF-1 in peripheral blood^21,64^. Perhaps, lifespan prolongation and delayed immunological and collagen senescence in mice with defects in production of growth hormone due to a homozygous mutation in the PROP1 gene (Ames dwarf mice) or Pit-1 gene (Snell dwarf mice) could be associated with the lack of multiple pituitary hormones (GH, PRL, TSH) not the isolated deficit of GH. The issue is exciting but requires further clinical study.

The limitations of our study include the relatively small sample size and the absence of a control group. However, it would be unethical to withhold GH treatment from GHD patients, as this would deprive them of the opportunity to improve their final height. Another limitation is the lack of MSC measurements after eight years of rhGH therapy; therefore, our conclusions regarding the long-term effects of rhGH on MSCs remain speculative.

In summary, long-term rhGH therapy modulates the populations of VSELs, MSCs, and EPCs circulating in PB. Our data confirm that VSELs respond to GH treatment and that long-term therapy does not compromise the naturally occurring VSEL population in GHD patients. Since rhGH modulates but does not deplete VSELs, it is unlikely—unlike in some experimental animal models—to negatively impact lifespan or organ rejuvenation in humans. These findings support an important role for VSELs in GH-regulated growth processes, in which HSCs contribute to body size expansion. Moreover, our data suggest that GH treatment may influence aging and rejuvenation in GHD patients, potentially through insulin signaling pathways rather than solely via IGF-1.

## Data Availability

The presented data and material are available from the authors of the article in the institutions where they the study was conducted. Anna Wędrychowicz (anna.wedrychowicz@uj.edu.pl) and Katarzyna Sielatycka (kasia.sielatycka@gmail.com) should be contacted if someone wants to request the data from this study.
